# The *in vivo* effect of Lacto-N-neotetraose (LNnT) on the expression of type 2 immune response involved genes in the wound healing process

**DOI:** 10.1038/s41598-020-57860-8

**Published:** 2020-01-22

**Authors:** Behrouz Farhadihosseinabadi, Mazaher Gholipourmalekabadi, Maryam Salimi, Mohammad-Amin Abdollahifar, Mohammad Bagheri, Ali Samadikuchaksaraei, Hossein Ghanbarian, Masoud Mozafari, Bahram Kazemi, Hassan Niknejad

**Affiliations:** 1 Department of Medical Biotechnology, School of Advanced Technologies in Medicine, Shahid Beheshti University of Medical Sciences, Tehran, Iran; 2 Cellular and Molecular Research Centre, Iran Universityof Medical Sciences, Tehran, Iran; 3 Department of Tissue Engineering & Regenerative Medicine, Faculty of Advanced Technologies in Medicine, Iran University of Medical Sciences, Tehran, Iran; 4 Department of Biology and Anatomical Sciences, Faculty of Medicine, Shahid Beheshti. University of Medical Sciences, Tehran, Iran; 5 Medical School, Shahid Beheshti University of Medical Sciences, Tehran, Iran; 6 Cellular and Molecular Biology Research center, Shahid Beheshti University of Medical Sciences, Tehran, Iran; 7 Department of Pharmacology, School of Medicine, Shahid Beheshti University of Medical Sciences, Tehran, Iran

**Keywords:** Reverse transcription polymerase chain reaction, Regenerative medicine

## Abstract

Lacto-n-neotatraose (LNnT) oligosaccharide shows properties such as anti-inflammatory, type 2 immune response induction, induced angiogenesis, and anti-bacterial effects. Here, we hypothesized that the application of LnNT in the skin full-thickness wound can accelerate the healing process through its anti-inflammatory effect as well as induction of type 2 immune responses. In this study, we evaluated the cell viability of fibroblasts in the presence of LNnT. The full-thickness wound model was created by punch biopsy. The mice were treated intradermaly with LNnT at the concentrations of 100 and 200 µg or PBS as a control group. The wounds samples were compared based on the macroscopic and histological evaluations. The amount of collagen deposition and expression of genes involved in type 2 immunity were measured by the hydroxyproline assay and real time PCR method, respectively. Our results showed that LNnT had no negative effect on the cell viability of fibroblasts. LNnT increased the wound closure rate on day 7 post-wounding. H&E stain analysis revealed that mice treated with 200 µg LNnT exhibited better healing score, follicle formation, and lower epidermal thickness index. The mice treated with LNnT exhibited a lower collagen deposition on day 21 and higher collagen content on days 7 and 14 post-treatment. The LNnT groups also exhibited a lower number of neutrophils and a higher number of basal cells and fibroblasts. The expression rate of IL-10, IL-4, and IL-13 was higher in the LNnT groups. These results showed the high potential of LNnT for use in treatment of full-thickness wounds.

## Introduction

Wound healing process is one of the well-known physiological mechanisms in which the body recruits various cells and growth factors to repair the damaged area. This process, as a fundamental mechanism, plays a pivotal role to keep the body hemostasis via avoiding the loss of water content as well as the entrance of pathological agents^1^. The great potential of healing mechanism in restoring skin function is enough for superficial wounds. However, in some cases such as full-thickness wounds, burns, and diabetic ulcers, the natural healing process is unable to fully recover the damaged site. In these circumstances, the medical interventions can be very helpful to accelerate the healing process^2^. At the early phase of wound healing, the cells involved in the inflammatory response come to the wound area. This phase is necessary to avoid post-wounding infections and help the damaged tissue regain its natural function. However, the continued activity and entry of inflammatory cells into the wound bed has a negative impact on the healing process^3^. In recent years, plenty of studies have been conducted to evaluate the potential effect of anti-inflammatory materials on the skin wound healing^4–6^. Lacto-N-neotetraose (LNnT) is a human milk-derived oligosaccharide which is also found in soluble egg antigens of *Schistosoma mansoni*^7^. This substance exhibits a wide variety of characteristics including anti-inflammatory effects^7,8^, type 2 immune response induction^9^, as well as anti-bacterial^10^ and angiogenic effects^11^, making it a suitable candidate for wound dressings. Type 2 immune responses include a variety of cell-cell and cell-cytokine interactions that mostly provide a firewall against parasites invasion in the body^12^. One of the important outcome is the wound healing acceleration due to secretion of different cytokines and growth factors by the cells involved in type 2 immunity^13^. Therefore, induction of type 2 immune responses can be an interesting and effective strategy in the treatment of full-thickness wounds^14^.

Here, we hypothesized that the application of LNnT, as an anti-inflammatory agent and an inducer of type 2 immunity, may have potential effects on the wound healing process. In this study, we investigated the effect of LNnT in the treatment of full-thickness wounds in the mouse model as well as the expression of the genes involved in type 2 immune response through the healing process.

## Materials and Methods

### LNnT preparation

25 mg of LNnT powder was purchased from Carbosynth Company (High Street, Compton, United Kingdom). After adding 1 mL of 1 × PBS buffer, the final solution was obtained at a concentration of 25 μg/μl.

### Cell culture

Normal human dermal fibroblasts (NHDFs) were cultured in Dulbecco’s Modified Eagle Medium (DMEM) supplemented with 10% fetal bovine serum (FBS) and 1% penicillin–streptomycin. The cells were then incubated in a humidified incubator at 37 °C and 5% CO_2_. The culture media were replaced every three days and the cells were passaged once a week. In this study, the cells of 3rd passage were used for the MTT assay.

### Cell viability analysis

To evaluate the cell viability in the presence of LNnT, the MTT assay was performed. LNnT (200 µg) was added to FBS-supplemented DMEM medium and sterilized using a 0.2-mm filter. Fibroblasts (5 × 10^3^) were seeded in 96-well plates and 200 µL of DMEM with or without LNnT was added to each well. The plates were incubated in the humidified incubator at 37 °C and 5% CO2 for 24, 48, and 72 h. After incubation in the aforementioned time points, 20 µL of MTT solution (2 mg/mL) was added to each well and incubated for 4 h. Then, the media containing MTT were discarded and replaced with 150 µL of DMSO. The optical density (OD) was measured using an ELISA reader at 490 nm. The cell viability percentage of fibroblasts in the presence of LNnT was calculated based on the following formula (Eq. 1)^15,16^:

1 $${\rm{Cell}}\,{\rm{viability}}\, \% =\frac{[OD]Test}{[OD]Control}\times 100$$

The cell viability of control (the untreated group) was considered to be 100%.

### *In vivo* study

The experimental procedures in this study were approved by Ethic Research Committee of Shahid Beheshti University of Medical Sciences under the ethical code number of 1397.376. All methods were performed in accordance with the relevant guidelines and regulations of Shahid Beheshti University of Medical Sciences. Sixty Balb/c male mice were randomly selected and acclimatized to the lab conditions for one week before the experiments. On the surgery day, the mice were anaesthetized by intraperitoneal injection of ketamine and xylazine. Two symmetric full-thickness wounds with a 6-mm diameter^17^ were created by the punch biopsy method on the shaved dorsal area of each mouse.

We decided to evaluate LNnT in the concentrations of 100 and 200 µg in our study. Since, it is the first time in literature that LNnT is used for treatment of skin wounds, the mentioned doses were selected based on the used concentrations of LNnT in two different studies^9,11^.

Accordingly, the mice were divided into the three groups; including mice treated with 200 μg of LNnT, 100 μg of LNnT, and PBS, as the control group. A single dose of LNnT was injected intradermally into the skin bordering the wounds and into the wound beds based on the protocol described by Naik-Mathuria *et al*.^18^. The experiments were carried out in four time points including 3, 7, 14, and 21 days post-surgery.

### Macroscopic observations

The wound closure rate was assessed at the aforementioned time points. In the postulated times, the photographs of wound areas were captured by a digital camera. Wound areas were measured using Digimizer software version 4. 1. 1. 0, and the wound closure rate was obtained based on the following formula (Eq. 2);

2 $${\rm{Wound}}\,{\rm{closure}}\,{\rm{rate}} \% =\frac{({\rm{wound}}\,{\rm{area}}\,{\rm{at}}\,{\rm{day}}\,0-{\rm{wound}}\,{\rm{area}}\,{\rm{at}}\,{\rm{each}}\,{\rm{postulated}}\,{\rm{time}})}{{\rm{wound}}\,{\rm{area}}\,{\rm{at}}\,{\rm{day}}\,0}\times 100$$

### Histological examinations

Five mice were sacrificed on 3, 7, 14 and 21 post-treatment days for each time point. The tissue samples were collected for histological analysis so that the wound bed and adjacent normal skin were removed and fixed in 10% formalin at room temperature for 48 h. The dehydration process was proceeded by ethanol. Following embedding the dehydrated samples in the paraffin blocks, various slice sections were prepared with a 4 µm-diameter. The slide sections were stained using Hematoxilin & Eosin as well as Masson’s trichrome. Five slide sections were selected from each paraffin block and 10 non-overlapping microscopic field-of-views were considered for all histological evaluations. The H&E-stained sections were analyzed for epidermal thickness index (ETI), new hair follicle formation, and wound healing scoring^19^. The collagen deposition was also determined through Masson’s trichrome staining. Three blind observation methods were used to count and score all evaluations.

### Epidermal thickness index (ETI)

H&E-stained sections were observed under a light microscope (Olympus BX51, Olympus, Tokyo, Japan) at 1000 magnification. To calculate ETI, the average thickness of epidermis areas from five randomly-selected fields were measured for both injured and uninjured skin sites. ETI was obtained based on the following Equation formula (Eq. 3).

3 $${\rm{Epidermal}}\,{\rm{thickness}}\,{\rm{index}}\,({\rm{ETI}})=\frac{{\rm{height}}\,{\rm{of}}\,{\rm{epidermis}}\,{\rm{in}}\,{\rm{injured}}\,{\rm{skin}}({\rm{scar}}\,{\rm{tissue}})\,}{{\rm{height}}\,{\rm{of}}\,{\rm{epidermis}}\,{\rm{in}}\,{\rm{normal}}\,{\rm{skin}}}$$

According to the mentioned formula, ETI of 1 indicates fully-healed wound without any scar formation and ETI >1 exhibits the hypertrophied epidermis formation.

### Hair follicles formation

To evaluate the hair follicle formation, 10 random fields of each H&E-stained section were observed under the light microscope and the average number of hair follicles was counted for each group. We then evaluated the hair follicle formation from the edge of wounds to the center area, where any new follicles were counted.

### Wound healing scoring

The wound healing score was measured according to the method described by *Gholipourmalekabadi et al*.^19^. Briefly, four scores from 1 to 4 were allocated to the quality of the healing process. Score “1” represents an immature granulation tissue filled by inflammatory cells and few fibroblasts. Score “2” indicates a relatively thick granular tissue with the significant number of inflammatory cells, more fibroblasts, and collagen content. Extensive neovascularization is also observed in this stage. Score “3” represents a thickened vascular granulation tissue, abundant fibroblasts and collagen deposition. Score “4” refers to the fully-healed wounds.

### Stereological study

The optical dissector method and H&E stain were used to determine the number of neutrophils, fibroblasts, and epidermal basal cells. To calculate the number of skin cells, 25-µm sections were used and 10 sections were selected randomly. The entire field of view of each section was evaluated using the Nikon’s light microscope (the E 200 model). According to this method, only those cells were counted that their nuclei were within the counting frame or on the acceptable lines. It should be mentioned that fibroblast, neutrophil and basal cells were distinguished based on morphological properties. Fibroblasts are spindle-shaped characterized by basophilic cytoplasm using H&E staining. Neutrophils have an approximately 6–9-µm diameter. They possess a single multilobed nucleus which has 2–5 lobes. A basal cell is a cuboidal-shaped stem cell that is a precursor of keratinocytes of epidermis with a round nucleus. The cells number was eventually obtained according to the following formula (Eq. 4);

4 $${\rm{N}}{\rm{\upsilon }}=\frac{{\sum }_{{\rm{i}}}^{{\rm{n}}}=1\,{\rm{Q}}}{{\rm{h}}\times {\sum }_{{\rm{i}}}^{{\rm{n}}}=1\,{\rm{p}}\times {\rm{a}}/{\rm{f}}}\times \frac{{\rm{t}}}{{\rm{AB}}}$$

$({\sum }_{i}^{n}\,=\,1Q)$ = Total number of all counted cell types

$({\sum }_{i}^{n}\,=\,1p)$ = Total number of the points superimposed on the selected fields

$$h$$ = Dissector height

*a*/*f* = Frame area in the actual tissue scale

t = Dissector practical thickness

AB = Dissector theoretical thickness

The result of equation was then multiplied by the total volume of the skin sample to obtain the total number of cells;

$${\rm{N}}({\rm{toti}})={\rm{N}}{\rm{\upsilon }}\times {\rm{V}}({\rm{reff}})$$

### Evaluation of hydroxyproline content

The hydroxyprolin assay was used to determine the collagen amount in the wound samples based on the Kiazist commercial kit instructions. The content of hydroxyprolin was evaluated per milligaram of dry specimen, according to a standard curve^20^.

### Real time PCR

Tissue samples from the wound sites were obtained at the aforementioned time points. Total RNA extraction and cDNA were synthesized using RNeasy Mini Kit (Cinagen, Tehran, Iran) and Hyperscript TM first strand synthesis kit (Geneall Biotechnology, Seoul, South Korea), respectively. The relative expression levels of IL-10, IL-4, and IL-13, as the main type 2 immunity-related cytokines, were determined by ABI Step One System (Applied Biosciences, Foster City, CA) using 2 × q PCR Master Mix Green-High Rox (Geneall Biotechnology, Seoul, South Korea). Beta-2 microglobulin (β2M) was used as a housekeeping gene to normalize the relative transcript level of each target gene. The relative expression level fold was identified based on the 2^−ΔΔCT^ method.

### Statistical analysis

For all variables, the Kolmogorov-Smirnov normality test was used for three groups using SPSS 16 software. According to their P-values, the groups were normal and, therefore, the parametric ANOVA tests with Tukey’s post-test were used to compare the group means. All data are represented as mean ± SD and p ≤ 0.05 was considered to be statistically significant.

## Results

### *In vitro* analysis and macroscopic observation

#### Cell viability analysis

**Figure 1:**
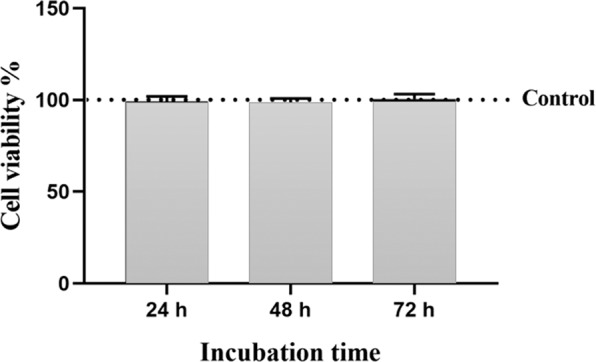
The MTT assay result; cell viability of control group is considered as 100%. There was no significant difference between the cell viability of LNnT and control groups in each time points.

Results from the MTT assay showed that the cell viability of fibroblasts after 24, 48, and 72 h in mice treated with LNnT was not significantly different from that in the control group (Fig. 1).

### Macroscopic evaluation and wound closure rate

**Figure 2:**
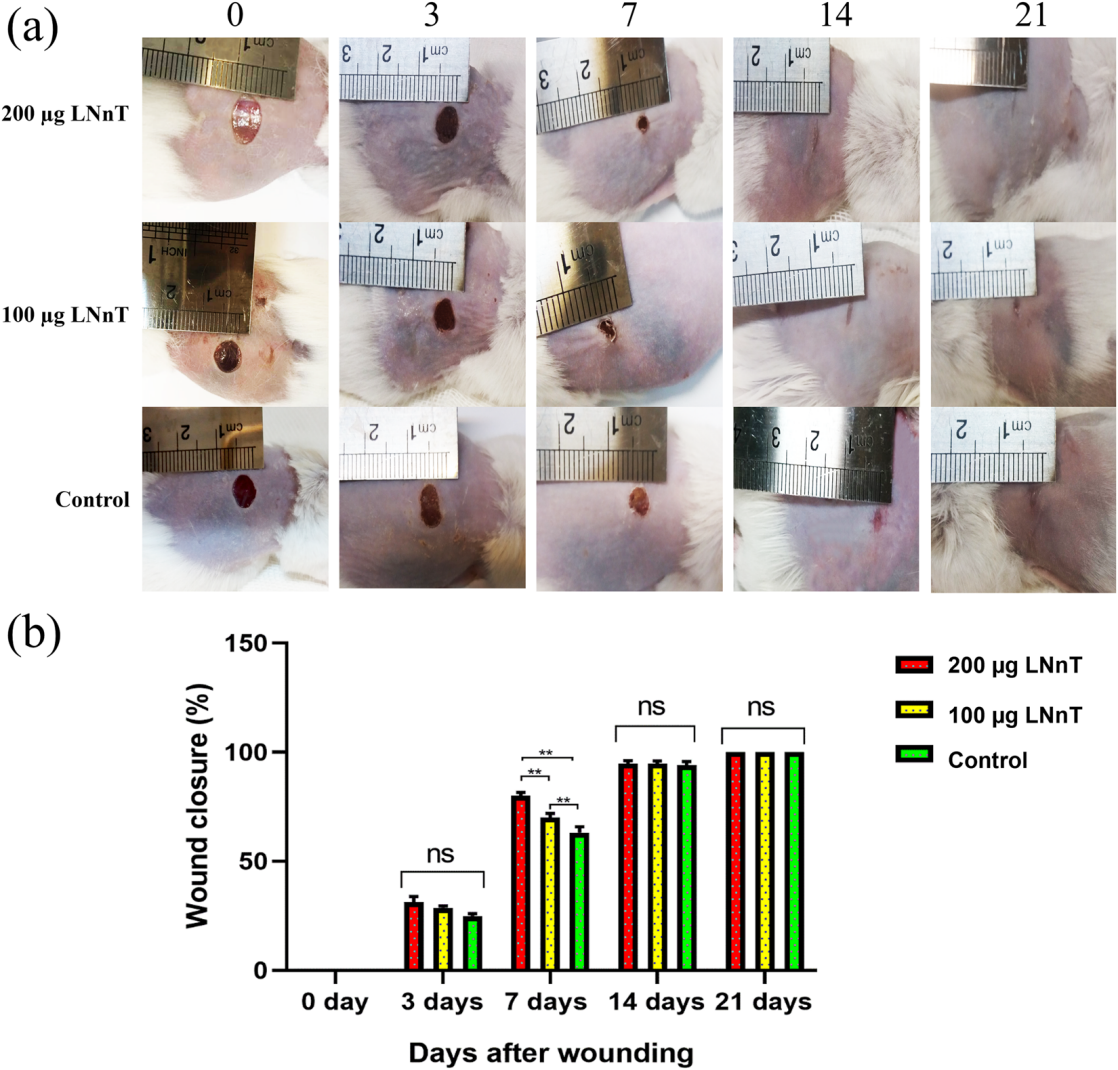
(**a**) Macroscopic observation of full-thickness wounds at 0, 3, 7, 14, and 21 days post-surgery. (**b**) Wound closure rate of full-thickness wounds at the aforementioned time points. ns: Non-significant; **P ≤ 0.01.

Figure 2a represents the photographs of wounds at 0, 3, 7, 14, and 21 days post-wounding. As shown in Fig. 2a, a significant difference was observed between wound size in mice threated with 200 µg LNnT and other two groups on day 7. Mice treated with 100 µg LNnT also showed better wound closure than the control on day 7. Since the wound closure was completed on days 14 and 21 in all experimental groups, evaluation of this parameter on day 7 could indicate the positive effect of LNnT in the wound closure.

The evaluation of wound closure rate using Digimizer software confirmed the macroscopic observations, where mice treated with 200 µg LNnT exhibited a significant difference compared with the 100 µg LNnT and control groups on day 7 (P < 0.0001). The same difference was also observed in mice treated with 100 µg LNnT compared with the control group, indicating the accelerating effect of LNnT on wound closure (Fig. 2b).

### Histological examinations

#### Hematoxylin and eosin (H&E) stain

**Figure 3:**
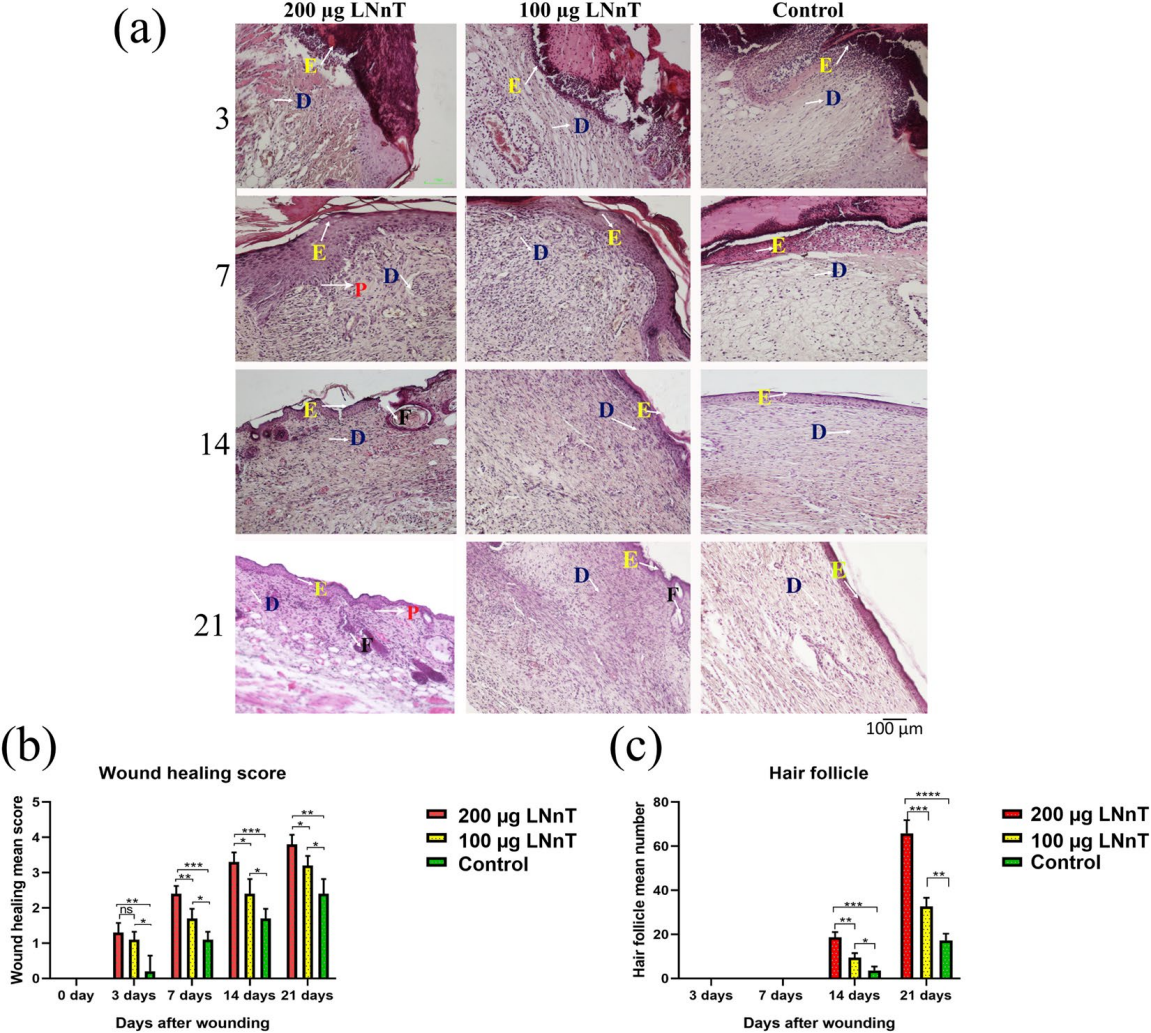
(**a**) H&E stained wound samples at the postulated time points. (**b**) Wound healing scoring. (**c**) Mean hair follicle formation. P: Rete pegs; D: Dermis; F: Follicle; E: epidermis layer. ns: Non-significant; *P ≤ 0.05; **P ≤ 0.01; ***P ≤ 0.001; ****P ≤ 0.0001.

Figure 3 represents H&E-stained wound sections on days 3, 7, 14, and 21 post-wounding. On day 3, re-epithelization was initiated gradually from the wound margin toward the wound center in all experimental groups. However, re-epithelialization in mice treated with 200 µg LNnT was found to be better than the other two groups. Moreover, cell infiltration (mostly polymorphonuclear cells) was clearly observed in all groups. Wound healing score in mice treated with 200 µg and 100 µg LNnT was significantly higher than the control group. Granular tissue formation was initiated in all experimental groups. However, the extracellular matrix deposition and granulation were clearly higher in mice receiving 200 and 100 µg LNnT. No hair follicles were observed in all groups at this point of time.

On day 7, the epithelial layer was completely formed in mice treated with 200 µg LNnT, which was larger in diameter than the other two groups. The epithelialization in mice receiving PBS was occurred incompletely. The amount of infiltrated cells in the wound bed was obviously higher in mice treated with 100 µg and 200 µg LNnT compared with the control group. In addition, granular tissue formation in mice treated with 100 µg and 200 µg LNnT was higher than in the control group. Wound healing score revealed a significant difference between mice treated with 200 µg LNnT and those treated with both 100 µg LNnT and PBS, which showed a better healing in mice receiving 200 µg LNnT. At this time point, no hair follicles were detected in all experimental groups.

On day 14 post-surgery, all wounds in the three groups were completely healed and re-epithelization process was successfully occurred in all experimental groups. As shown in the Fig. 3a, the amount of rete pegs in mice treated with 100 µg and 200 µg LNnT were clearly higher than the control group, demonstrating a better repair and less scar formation. Wound healing evaluation exhibited a better score for mice receiving LNnT compared with the control. Moreover, hair follicle formation in mice treated with 200 µg LNnT was significantly higher than those treated with 100 µg LNnT as well as the control group.

On day 21 post-wounding, the structure and appearance of wounds in mice treated with 200 µg LNnT were closely similar to the normal skin, representing a higher number of hair follicles and rete pegs as well as a lower cellularity and granulation when compared with the other groups. Moreover, in this point of time, wounds in mice treated with 200 µg LNnT showed the highest wound healing score compared with those treated with 100 µg LNnT as well as the control group. The wound healing evaluation in mice treated with 100 µg LNnT also exhibited a better healing score than the control group, which indicates the positive effect of LNnT in the wound healing process.

#### Epidermal thickness index

**Figure 4:**
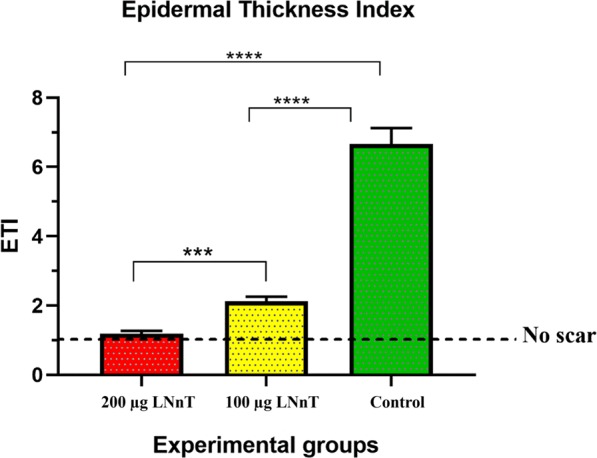
Epidermal index results (ETI) on day 21 post-surgery. ***P ≤ 0.001; ****P ≤ 0.0001.

On day 21 post-surgery, ETI of all wounds was measured using Eq. 3. Our results showed that the diameter of epidermal layer in mice receiving 200 µg and 100 µg LNnT was close to the normal skin, while the ETI of control group was remarkably higher than normal skin (Fig. 4).

#### Masson’s trichrome stain

**Figure 5:**
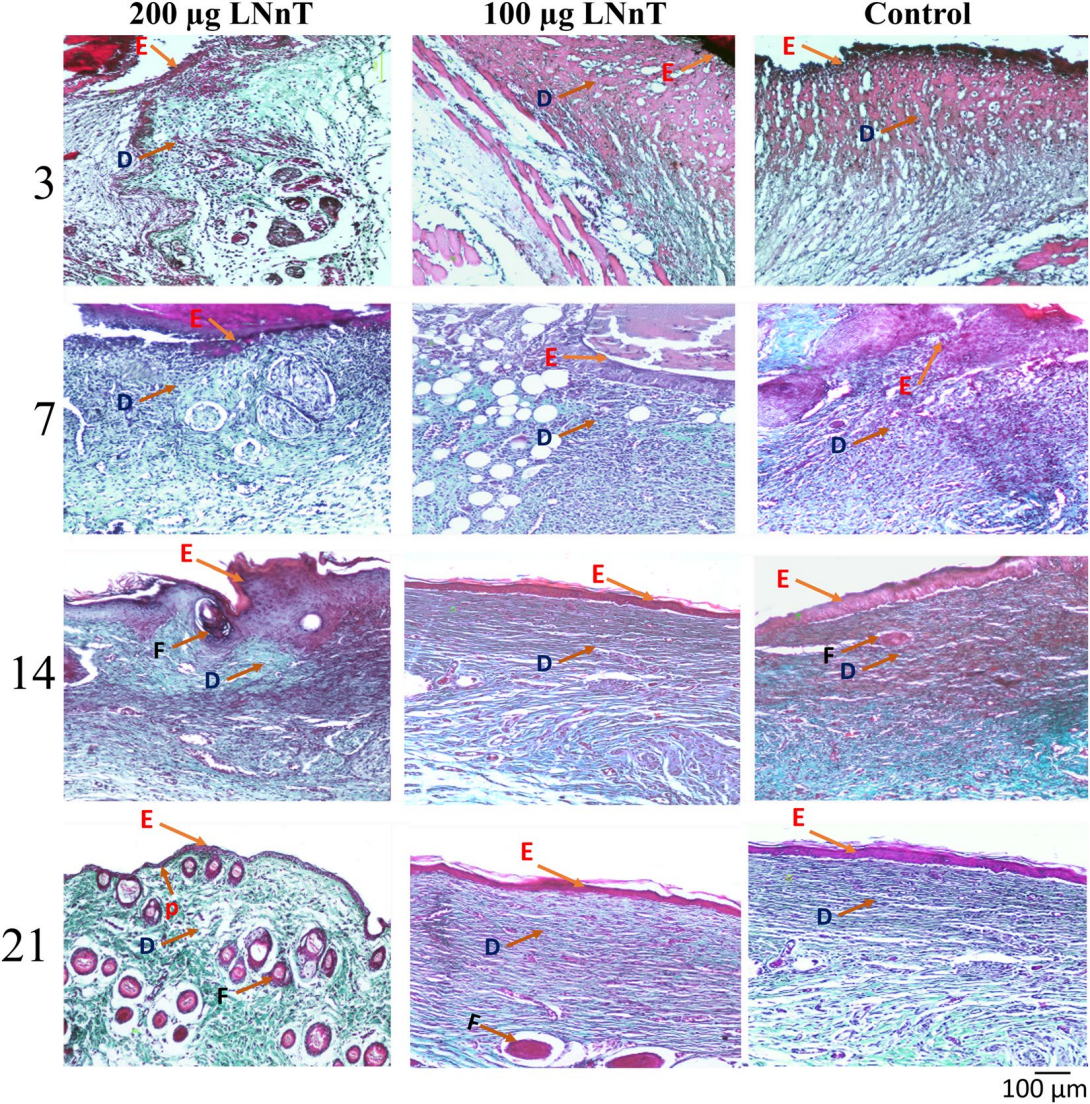
Masson’s trichrome staining of wounds samples at 3, 7, 14, and 21 days post-wounding. P: Rete pegs; D: Dermis; F: Follicle; E: epidermis layer.

Collagen deposition in all Masson’s trichrome stained samples was evaluated at days 3, 7, 14, and 21 post-surgery (Fig. 5). On day 3, the samples of mice treated with 100 µg and 200 µg LNnT showed a small amount of collagen synthesis in the wound area, which was not clearly observed in the control group. Collagen content in mice treated with 200 µg LNnT was significantly higher than those treated with 100 µg LNnT as well as the control group on day 7 post-wounding. Moreover, there was a difference in collagen deposition of mice treated with 100 µg LNnT and the control group in this point of time. It showed the potential of LNnT in provoking collagen synthesis within the wound area. On day 14, the collagen deposition rate in mice receiving LNnT was higher than that in the control group. On day 21, collagen deposition in mice treated with 200 µg LNnT was almost similar to the normal skin around the wound, while the collagen content in the control group was significantly higher than that in the normal skin. Our results showed that LNnT could effectively accelerate the wound healing process and decrease the scar formation.

#### Stereological study

**Figure 6:**
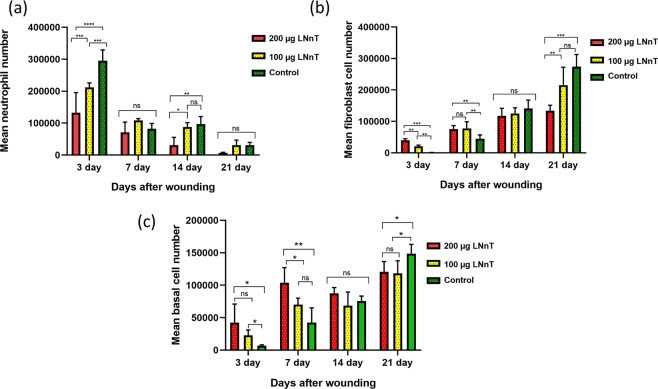
Stereological results of neutrophil (**a**), fibroblast (**b**), and basal cells (**c**) number in the wound samples at 3, 7, 14, and 21 days post-wounding. ns: Non-significant; *P ≤ 0.05; **P ≤ 0.01; ***P ≤ 0.001; ****P ≤ 0.0001.

The stereological experiment was carried out in order to compare the cells number in the wound bed. The mean neutrophil, fibroblast, and basal cells were compared between 3 groups at 3, 7, 14, and 21 days post-wounding. Our results showed that LNnT could reduce the neutrophil number in the wound bed (Fig. 6a). On day 3, mice receiving LNnT exhibited a lower number of neutrophils compared with the control.

Fibroblasts play a major role in the production of new extracellular matrix within the wound bed. The stereological results demonstrated that mice receiving LNnT show a higher number of fibroblasts at 3 and 7 days post-surgery (Fig. 6b). Interestingly, fibroblast counts on day 21 in these animals were significantly lower compared with those receiving no LNnT treatment. These data confirmed the masson’s trichrome staining results that showed a lower collagen deposition in mice treated with 100 µg and 200 µg LNnT on day 21 post-wounding.

Proliferation of epidermal basal cells is associated with increased re-epithelialization and wound healing^21^. Our stereological study showed that LNnT could successfully increase the number of basal cells in the wound bed (Fig. 6c). According to our results, the number of basal cells on day 3 post-wounding was significantly higher in mice treated with 100 µg and 200 µg LNnT than the control group. Moreover, on day 7, mice receiving 200 µg LNnT exhibited a higher number of epidermal basal cells compared with those treated with 100 µg and PBS.

### Hydroxyproline content evaluation

**Figure 7:**
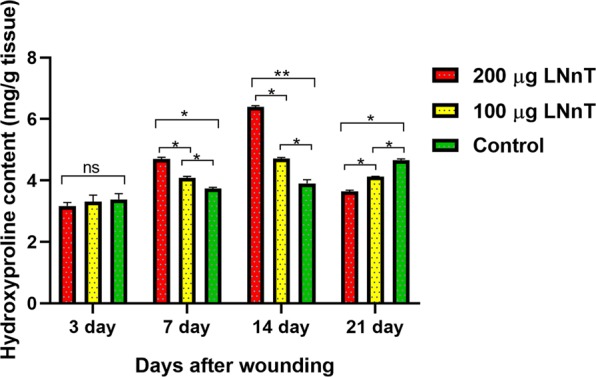
The hydroxyproline content of the wound samples at 3, 7, 14, and 21 post-wounding. ns: Non-significant; *P ≤ 0.05; **P ≤ 0.01.

The hydroxyproline assay was performed to find the capability of LNnT in alteration of collagen content within the wound bed. In this assay, the level of hydroxyproline is directly correlated with the collagen deposition rate, making it an appropriate marker for evaluation of collagen amount. Our results showed that the hydroxyproline level at 3 days after wounding was not significantly different in the all three groups. On days 7 and 14 post-wounding, the amount of hydroxyproline in the groups receiving LNnT was significantly higher than control, indicating the potential of LNnT in increasing the collagen deposition. The level of hydroxyproline at day 21 post-wounding was lower in the groups treated with LNnT compared with the control group (Fig. 7).

### Gene expression profiles

**Figure 8:**
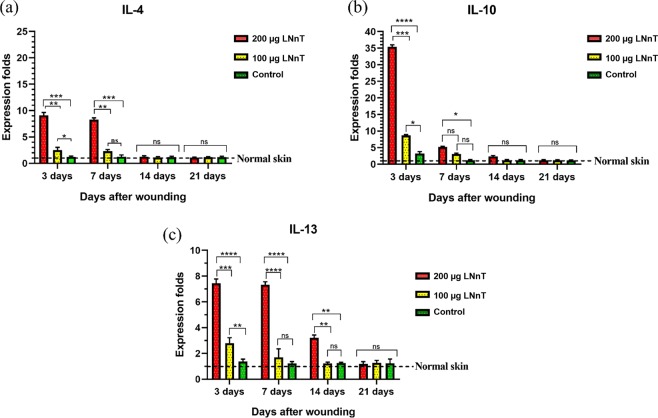
Real-time PCR results of relative expression of IL-4 (**a**), IL-10 (**b**), and IL-13 (**c**) at 3, 7, 14, and 21 days post-surgery. All expression rate of the targeted genes were normalized to that in normal skin.

Real time PCR was used to evaluate the effect of LNnT on gene expression profile of wound samples. The expression rate of the three major genes associated with type 2 immune response, including IL-4, IL-10, and IL-13, were measured in the wound samples. Our results showed that the expression rate of IL-4 on day 3 post-treatment was significantly higher in mice treated with 200 µg LNnT than those treated with 100 µg LNnT as well as the control group (Fig. 8a). This difference was also maintained during the first week after wounding. However, the significant difference between mice treated with 100 µg LNnT and control groups on day 3 was not observed on day 7 post-surgery. However, the expression rate of IL-4 on days 14 and 21 was slightly higher in mice receiving LNnT than in the control group; this difference was not statistically significant.

The highest expression rate of IL-10 was found among the tested genes on day 3 post-wounding (Fig. 8b). In this point of time, mice treated with 200 µg LNnT exhibited a significant difference in the IL-10 expression rate compared with the other groups. Moreover, mice treated with 100 µg LNnT showed a higher expression rate of IL-10 than the control group. These data showed the significant effect of LNnT in the induction of IL-10 production in the early stage of the inflammation phase. On day 7, the expression of this gene in mice treated with both 100 µg and 200 µg LNnT significantly decreased compared with those on day 3, but the expression of this gene in mice treated with 200 µg LNnT was still higher than that of treated with 100 µg and the control group.

Figure 8c showed the expression rate of IL-13 in all experimental groups at the aforementioned time points. According to our results, the expression level of this gene in the animals treated with 200 µg LNnT was remarkably higher than those treated with 100 µg LNnT as well as the control group. This difference was also observed on days 7 and 14 post-treatment. The mice treated with 100 µg LNnT showed a statistical difference when compared with the control group only on day 3 post-wounding.

## Discussion

Full-thickness wound is one of the most common type of skin damages. Delayed wound healing process in this skin lesion may lead to some serious consequences including post-injury infections and hypertrophic scars^22,23^. Therefore, researchers are trying to apply different strategies to accelerate the repair process and prevent these consequences. Reducing inflammation within the wound area is one of the effective strategies to accelerate the wound healing process^24^. Pathogens entrance into the damaged skin calls inflammatory cells such as neutrophils. The inflammatory cells secrete various proteases and ROS to inhibit infections and bacterial colonization. In the acute or prolonged inflammation, over-production of anti-bacterial agents secreted by these cells leads to degradation of growth factors and extracellular matrix within the wound bed which consequently impair wound closure and healing^25–28^. Moreover, reducing inflammation provides an appropriate balance in the production of new extracellular matrix proteins at the granulation phase of the healing process which eventually decrease the scar tissue formation^29,30^. Until now, various materials with the anti-inflammatory effect have been successfully utilized in this context. For example, *Wilgus et al*.^31^ examined the effect of celecoxib, as a known anti-inflammatory drug, on full-thickness wounds in the mouse model. According to their reports, animals treated with celecoxib exhibited a lower amount of neutrophil infiltration into the wound bed. The animals also showed a lower collagen deposition which consequently led to the less scar formation when compared with the control. As mentioned above, LNnT possesses an anti-inflammatory potential which make it a suitable candidate in the wound treatment. Our results showed that mice treated with LNnT exhibited a smaller wound size at the macroscopic level. Beside this, the H&E analysis indicated that mice receiving LNnT had better wound healing score, follicle number, and ETI compared with the control. They also possessed a higher number of basal cells and fibroblasts. Results from the Masson’s trichrome stain also indicated the anti-scar potential of LNnT in the wound healing process where the animals receiving LNnT exhibited a lower collagen deposition on day 21 post-surgery.

*Bode et al*.^32^ reported the positive effect of human milk-derived oligosaccharides (HMOs) on decreasing neutrophil adhesion to the endothelial cells. They reported that HMO-treated leukocytes showed a lower attachment to the HUVEC cells. The leukocyte attachment to the endothelial cells is an essential stage for leukocyte rolling and infiltration to the desired site. Recently, an investigation has revealed that HMOs acts as a soluble selectin ligand analogs which blocks the interaction between p-selectin on the platelets and PSGL-1 on the neutrophils. The interaction between these two selectins provides the early step of platelet-neutrophil-complex formation, resulting in activation of signaling cascade that increase the expression of adhesion molecules on the neutrophils. Therefore, any intervention that reduces the formation of the complex can diminish the rate of neutrophil infiltration into the inflamed tissue^33^. Our results were in line with the mentioned studies as a significant reduction was observed in the neutrophil infiltration into the damaged site in mice treated with 100 µg and 200 µg LNnT at the early inflammation stage of wound healing process.

To evaluate the anti-inflammatory effect of LNnT within the wound bed at the molecular level, we analyzed the expression level of IL-10, as a main anti-inflammatory cytokine, in the wound samples. Our results showed an increased expression rate of IL-10 in both LNnT-treated groups. It was reported that the overexpression of this cytokine is associated with the decreased scar formation^3,34^. In fact, IL-10, in addition to the anti-inflammatory effect, has the ability to regulate the production of fibrogenic cytokines that governs its regulatory role in the tissue remodeling phase of wound healing^35^. In our study, there was a direct correlation between increased expression of IL-10 and decreased inflammation and collagen deposition in animals receiving LNnT. These data showed that LNnT can provoke the immune system to produce a large amount of IL-10 in the wound bed. Therefore, as an anti-inflammatory agent, LNnT can be a suitable candidate to be applied in the wound care system. Moreover, the anti-bacterial effect of the LNnT may also diminish the inflammation within the wound bed. For instance, LNnT can effectively block the adherence of bacteria to the epithelial cells, resulting in inhibition of bacterial colonization^36^. Decreased bacterial colonization reduces inflammation in the wound area which may be another reason for anti-inflammatory effect of LNnT.

As mentioned above, the induction of type 2 immune response activates a cascade of events promoting a firewall against pathogens, especially parasites. One the most important outcome of this type of immune response is the accelerated wound healing process.

LNnT, as a main component of parasite antigens, was reported to have a key role in provoking type 2 immune response in the body^13^. Terrazas *et al*.^9^ showed that the peritoneal injection of 100 µg of LNnT-Dextran in mice could expand and proliferate the cells involved in type 2 immunity such as Gr1^+^/CD11b^+/^F4/80^+^. Moreover, the co-culture of naïve T-cells with peritoneal exudate cells (PECs) obtained from animals receiving LNnT-Dextran exhibited an increased production of IL-13 and IL-4 compared with those obtained from dextran-treated and non-injected mice. In addition, naïve T-cells were polarized and showed a Th-2 phenotype in the presence of PECs obtained from animals receiving LNnT-Dextran. It is reported that IL-4 and IL-13 activate fibroblasts and force them to produce higher amount of ECM proteins^37,38^. *Wynes et al*. reported that IL-4 and IL-13 cytokines promote macrophages to produce insulin-like-growth factor-I that possess the inhibitory effect on myofibroblast apoptosis^39^. In the present study, we examined the effect of LNnT in expression of two Th-2 related cytokines, IL-4 and IL-13, in the wound samples. Our results showed that the expression rate of IL-4 and IL-13 was significantly higher in the wounds treated with LNnT compared with the control. It can explain why the collagen deposition in the groups treated with LNnT was higher than the control. It seems that LNnT could alter the immune response in wounds toward type 2 immunity. However, the recent claim needs to be further investigated to confirm. In the current study, we evaluated the cytokines involved in type 2 immune response only at the gene expression level. It is suggested that the evaluation of IL-4, IL-10, and IL-13 within the wound area at the protein level will further confirm the role of type 2 immunity in the LNnT-induced wound healing.

TGF-β is an important cytokine in the wound healing process. However, the role of TGF-β in reducing inflammation mediated by milk-derived oligosaccharides is controversial. For example, Grabinger *et al*.^40^ reported that milk oligosaccharides as a food supplement could significantly increase the TGF- β in the mice with colitis. This result was not in line with those obtained from Lara-Villoslada^41^ investigation, in which the milk oligosaccharides decreased inflammation in the dextran sodium sulfate- (DSS-) induced rat colitis model in a TGF-β independent manner. We suggest that evaluation of TGF- β in the future studies would help us to find out the role of TGF-β signaling in skin wound healing mediated by LNnT.

## Conclusion

In the present study, we evaluated the effect of lacto-n-neotetraose in the healing process of the full-thickness wounds in the mouse model. Our results showed that LNnT could remarkably improve the wound closure, wound healing score, follicle number, ETI and collagen deposition (on day 7) compared with the control. Moreover, LNnT decreased the collagen deposition (on day 21) as well as scar formation in the wound samples. In the molecular level, LNnT increased the expression of anti-inflammatory cytokine, IL-10, and also elevated the expression rate of two type 2 immune response-involved genes including IL-4 and IL-13. These results indicate the positive effect of LNnT oligosaccharide in the skin wound healing process which makes it an appropriate material for the skin wound care system.
